# Energy Efficient Multipath Routing Algorithm for Wireless Multimedia Sensor Network

**DOI:** 10.3390/s19173642

**Published:** 2019-08-21

**Authors:** Addisalem Genta, D. K. Lobiyal, Jemal H. Abawajy

**Affiliations:** 1 Department of Electrical and Computer Engineering, Institute of Technology, Ambo University, Ambo, Ethiopia; 2 School of Computer and System Sciences, Jawaharlal Nehru University, New Delhi-110067, India; 3 Faculty of Science, Engineering and Built Environment, Deakin University, Geelong, VIC 3125, Australia

**Keywords:** WMSN, energy efficiency, GA, multipath, routing, network lifetime

## Abstract

Wireless multimedia sensor networks (WMSNs) are capable of collecting multimedia events, such as traffic accidents and wildlife tracking, as well as scalar data. As a result, WMSNs are receiving a great deal of attention both from industry and academic communities. However, multimedia applications tend to generate high volume network traffic, which results in very high energy consumption. As energy is a prime resource in WMSN, an efficient routing algorithm that effectively deals with the dynamic topology of WMSN but also prolongs the lifetime of WMSN is required. To this end, we propose a routing algorithm that combines dynamic cluster formation, cluster head selection, and multipath routing formation for data communication to reduce energy consumption as well as routing overheads. The proposed algorithm uses a genetic algorithm (GA)-based meta-heuristic optimization to dynamically select the best path based on the cost function with the minimum distance and the least energy dissipation. We carried out an extensive performance analysis of the proposed algorithm and compared it with three other routing protocols. The results of the performance analysis showed that the proposed algorithm outperformed the three other routing protocols.

## 1. Introduction

Conventional wireless sensor networks (WSNs) consist of low-cost sensor nodes that are capable of sending scalar physical phenomena (e.g., temperature, pressure, and humidity), necessary computation, and transmitting data wirelessly [1]. New generation multimedia sensor nodes are capable of pervasively capturing multimedia contents as well as scalar data from the sensing fields. The availability of multimedia sensor nodes coupled with the novel distributed signal processing [2] and multimedia source coding algorithms [3] have enabled the emergence of wireless multimedia sensor networks (WMSNs) that are capable of capturing, transmitting and receiving multimedia contents [4]. WMSNs support many civilian and military applications such as the detection of plant diseases [5], the detection of the presence of insects [6], wildlife tracking [7], situation awareness [8], security monitoring [9], and automatic threat classification [10]. This inherent ability to be deployed for a wide range of applications has made WMSNs an attractive platform for applications that need pervasive access to multimedia contents. As a result, WMSNs-are increasingly gaining traction in the research community as well as in industry.

In this paper, we address the problem of how to reduce energy consumption and routing overheads in WMSNs. One of the significant and essential requirements of WMSNs is the efficient transfer of multimedia data from a target node to the base station, taking into account several design considerations such as energy efficiency and fault tolerance. Minimizing energy consumption and overheads is an important issue in WMSNs because multimedia applications generally generate a high volume of network traffic. This traffic requires high transmission rates and processing capabilities from the nodes. Since both of these activities consume high energy, they will lead to premature node energy depletion and consequently shorten the WMSN network lifetime. Therefore, the design of an efficient and reliable routing protocol to maximize the network lifetime while simultaneously satisfying the quality of service requirements of the multimedia applications is critical in WMSNs. However, the inherent characteristics of WMSNs such as the variable channel capacity, limited bandwidth, and dynamic links pose severe challenges to routing protocol design for WMSNs. Furthermore, the limited resources of the sensor nodes such as energy, memory, buffer size, bandwidth, and processing capability all pose additional challenges to routing algorithm design for WMSNs. Moreover, the characteristics and requirements of multimedia applications, such as high bandwidth demand and bounded delay, further exacerbate the routing protocol design for WMSNs. Although many approaches have been proposed, the problem of a routing protocol design for WMSNs remains a challenge [11].

To address the aforementioned problem, developing a routing protocol that efficiently discovers and chooses a routing path between the sensing nodes and the sink, with low overheads, is very important in WMSNs. Routing protocols that employ a single path approach tend to be very sensitive to the highly dynamic nature of the wireless links and the limited capacity of the multi-hop path, which make them incapable of providing high data communication for WMSNs [12,13,14]. Moreover, the performance of the WMSN can be influenced profoundly by congestion [15,16] since the sensor nodes have limited resources like bandwidth, memory, and computational energy. As a result, many different types of routing protocols with various approaches for enhancing the efficiency of the routing protocols such as clustering, multipath routing, and multi-hop routing have been advanced in the literature [17]. These routing protocols are almost always based on maximizing energy efficiency, and rarely consider efficient routing path selection and routing overhead issues simultaneously. Therefore, without appropriate and careful routing of multimedia data in WMSNs, the performance can be affected and the lifetime of WMSNs can also be shortened.

In this paper, we propose an energy efficient multipath algorithm with dynamic clustering using genetic algorithm(GA) meta-heuristic optimization. Since finding optimal routes in a network is known to be an NP-complete problem [18], efficient heuristic search algorithms based on reduced-complexity are necessary. The algorithm uses two different methods, namely, clustering and multipath routing algorithms, to reduce energy consumption without unnecessarily impacting on the performance of the network. In the proposed approach, the sensor nodes in the network are first dynamically partitioned into clusters using a novel dynamic clustering algorithm. Then the cluster head for each cluster is selected using an efficient cluster head selection algorithm. Finally, an inter-cluster multipath routing is developed using the GA optimization algorithm to find the best path for the transmission of the sensed data. Since the proposed routing algorithm uses GA-based meta-heuristic optimization, it can select the best possible routing and has great ability to effectively deal with the dynamic topologies of WMSN.

Our contributions in this paper can be summarized as follows:

- We propose a novel algorithm for dividing the network into clusters each with a cluster head (CH) to save more energy and to improve network lifetime.
- We proposed energy efficient multipath algorithm with dynamic clustering using Genetic Algorithm (GA) based meta-heuristic optimization technique.
- We performed an extensive performance analysis of the proposed energy efficient multipath algorithm and compared it with several states of the art routing algorithms for WMSN

The rest of the paper is organized as follows. In Section 2, the related works are reviewed. In Section 3, the system model used in this study is presented. In Section 4, the proposed routing algorithm is presented. The experiment and the analysis are discussed in Section 5. The conclusion and future work are presented in Section 6.

## 2. Related Work

Routing algorithms for WMSNs is an active research area [15,17,19,20]. A pair-wise directional geographical routing was proposed to solve energy issue in multi-path routing for WMSNs [21]. The scheme improves the network lifetime by compromising on the delay time. A seamless streaming data delivery protocol was proposed in [22]. The scheme applies cross-cluster handover and path redirection strategies to minimize delivery latency and energy consumption. However, the performance deteriorates with an increase in the size of the sensor network, e.g., the addition of powerful multimedia sensor nodes. 

Multipath routing has been an attractive approach in MWSN for reducing latency and energy consumption as well as attaining high throughput. A multipath routing algorithm called direct diffusion (DD), which has a query driven basis, is discussed in [23]. The algorithm begins its process by broadcasting a packet. The node which acquires the packet establishes a connection with the sender node. Likewise, multiple paths will be created between every pair of source and sink nodes. If a node identifies the presence of an event, it automatically delivers the information to the sink. The best path among the multiple paths will be chosen by the sink node using packet reception performance metrics such as minimum latency in the case of data communication. Nevertheless, this routing mechanism is more suitable for applications which are query-driven and contains all the drawbacks of DD. The REAR protocol [24] addresses energy efficiency and reliability in terms of data delivery. The protocol maintains an alternative path to the sink node and thus supports multi-path reliable data transmission. The base station (BS) starts the routing process by broadcasting a service-path query to the other nodes in the network. After receiving the message, the nodes will send back a reservation message to the BS to make sure that a path is discovered. The performance of the protocol has been evaluated in comparison with other existing techniques. However, distance related parameters are not considered in the REAR protocol. A multipath routing algorithm with a minimum delay for WSN is discussed in [25]. Parameters such as the network lifetime during braided and disjoint multipath techniques were used as one of the performance indicators when the protocol was compared with single path routing techniques. The criteria for choosing the best path is a minimum hop count. The algorithm achieves the minimum delay as compared to other existing algorithms. However, the algorithm does not consider residual energy and the distance. 

An algorithm called EECA is discussed in [26]. EFCA discovers two different collision-free paths. Furthermore, EFCA adopts constrained and power adjusted flooding by the base station to reduce unwanted energy wastage of the system. Though the simulation results verified its efficiency in terms of energy saving and data transmission, there is still an opportunity to improve EFCA since collisions along multiple paths often occur. A protocol for a real-time application that is reliable and delay aware is discussed in [27]. Reliability is achieved via using forward error correction (FEC). The delay constraint is satisfied by the redundant data introduced during the data communication. Similar to other protocols, it uses a flooding technique for multiple path discoveries. The remaining energy of the sensor node, distance to the sink node, and path length were not analyzed in the study and can be considered as a limitation of this work. In [28], the authors discussed the advantage of employing a multipath routing technique to achieve reliable data delivery in a wireless sensor network based on hop count and ring level. The ring level is used to divide the nodes into several sections. During network setup, the base station floods the network with messages to investigate and confirms which nodes are members of a certain ring level. The ring level will increase whenever the hop count increases. Suppose a particular sensor node is member of ring level *n* and it sends *s* data to *n-1* ring level. This technique improved LEACH routing by 27.58% and direct routing by 113.06%. Since energy utilization and the lifetime of the network were not considered in the proposed work, these can be considered as future work. A multipath multispeed protocol (MMSPEED) [29] is proposed with the aim of guaranteeing correct delivery of packets in a WSN. The packets are queued in sequence based on their speed level. MMSPEED uses a priority service policy to service the queued messages. Those packets that have higher priority will be serviced first and packets with lower priority will be serviced last. Unfortunately, energy-related parameters and network lifetime maximization were not considered in this work.

An ant colony optimization (ACO)-based multipath routing algorithm is presented in [30]. Residual energy, transmission energy consumption, and length of the path were some of the metrics considered in the design. After CH is chosen in the cluster, ACO is applied to find multiple paths between CH and the base station. CH is dynamically selected to deliver data based on probability, which is a function of various parameters such as energy consumption. The proposed technique minimized the average energy consumption and maximized the network lifetime. Genetic algorithm (GA) is a meta-heuristic that is based on the theory of natural selection where the fittest chromosome will survive for reproducing new offspring in the next generation. Many researchers have used a genetic algorithm for node deployment, routing, and even clustering in wireless sensor networks [31,32,33,34,35]. In [36], a genetic algorithm for energy efficient routing is proposed, where GA is used for scheduling data gathering and aggregation of relay sensor nodes. The performance analysis shows that the approach finds an optimal solution for both small and large networks. In [37], a genetic algorithm is used to solve coverage and connectivity issues in WSNs. This method is used to find a few appropriate positions to deploy sensor nodes, satisfying both coverage and connectivity in the target area. The algorithm provides k-coverage to all targets and m-connectivity to each sensor node. The GA’s chromosome was represented efficientlyand the objective function was also derived properly with all operators. A GA-based clustering and routing approach for wireless sensor networks [38]. Distances from the nodes to their CH, along with the residual energy of gateways were taken as a decisive parameter for cluster formation. The routing protocol depends on the tradeoff between multiple re-transmissions of data, and transmission distance and the remaining energy of the gateways. Similarly, in this study, we employed GA for finding multiple paths in a wireless multimedia sensor network. Our scheme uses the advantage of GA’s computational efficiency to quickly find a solution to the problem. As a result, our GA-based approach can quickly compute an alternative new routing path from a source node to the sink node.

## 3. System Model

In this section, we briefly describe the system model, which comprises both the network and the energy models. Table 1 lists the symbols used throughout the paper.

### 3.1. Network Model

**Figure 1:**
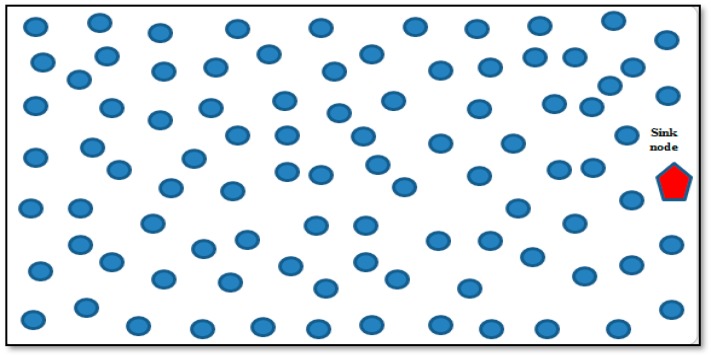
Wireless multimedia sensor network model.

The WMSN consists of n battery-powered multimedia wireless sensor (MWS) nodes and a BS, as shown in Figure 1. We use a weighted undirected graph G=(V, E, W) to represent WMSN where $V=\;\left\{v_{1},{\;v}_{2},{\;v}_{3},\;\ldots ,{\;v}_{N}\right\}$ is the vertex set representing the MWS nodes in the network, $E=\;\left\{e_{1},{\;e}_{2},{\;e}_{3},\;\ldots ,{\;e}_{n}\right\}$ represents the set of all bidirectional links and $W=\;\left\{w_{1},{\;w}_{2},{\;w}_{3},\;\ldots ,{\;w}_{n}\right\}$ is the set of weights on the links. Each MWS node, $v_{i}\in V$, consists of a sensing unit to monitor the environment, a processing unit to process information, a transceiver unit to transmit and receive the information wirelessly, and a power supply unit. The sensor nodes are randomly deployed in a square sensing field to monitor and detect, and collect information on dynamic events. BS collects information gathered by the MWS nodes to be stored or further processed.

Each multimedia wireless sensor has a unique ID, a communication radius, R, and a set of neighboring nodes defined as $N\left(v_{i}\right)=\left\{v_{j}/d\left(v_{i},v_{j}\right)\leq {R\;\mathrm{and}\;v}_{j}\varepsilon V\right\}$, where $d\left(v_{i},v_{j}\right)$ is the distance between the node $v_{i}{\;\mathrm{and}\;\mathrm{node}\;v}_{j}$. We assume that the n sensor nodes do not know their location, but they are capable of estimating the distance of their neighbor nodes. There exists a bidirectional wireless link, $e_{ij}\in E$, between two sensor nodes, $v_{i}\in V$ and $v_{j}\in V$, such that$\;v_{i}\neq v_{j}$ and$\;d\left(v_{i},\;v_{j}\right)\leq R$. The weight,${\;w}_{\mathrm{ij}}$, on a given link, $e_{ij}\in E$, denotes the energy consumed when data are being transmitted from node $v_{i}$ to node $v_{j}$, such that$\;v_{i}\neq v_{j}$.

The sensor nodes are battery-powered and assumed to be deployed in an area where it is very difficult to reach every node and replace their sensor batteries. In order to prolong the lifetime of the network, the energy consumed by each sensor node should be minimized. To address this problem, multimedia sensor nodes are organized into clusters using a clustering mechanism (Section 4.1) with each cluster containing a set of cluster members and a cluster head (CH). The main purpose of the CH is to coordinate the cluster activities and the CH is dynamically elected. The cluster members have direct access to only one CH. Thus, each communication initiated by a cluster member to a destination inside the cluster must pass by the CH. WMSNs also experience failures for a variety of reasons such as network hole problems [39,40], which have a great impact on the network during data transmission from source to destination. To handle this problem, we use a multipath routing algorithm as explained in Section 4. As noted earlier, the sensor nodes monitor dynamic events and, on detection, collect and forward the information to the cluster head. The event can be detected by a set of nodes, $S_{\mathrm{event}}=\;\left\{v_{1},{\;v}_{2},{\;v}_{3},\;\ldots ,{\;v}_{m}\right\}$, where m<n and${\;S}_{\mathrm{event}}\subseteq V$.

The performance of a WMSN can be profoundly influenced by congestion [16,20] since the sensor nodes have limited resources in terms of bandwidth, memory, and computational energy. Thus, a multipath routing approach can be utilized as a proper means to minimize the occurrence of network congestion by dividing the traffic into many independent paths [41]. Besides, sharing the network traffic load among several nodes can enhance the life span of the network by reducing the energy consumption of each node. It has also been proven that the multipath routing protocol improves the performance of the wireless sensor network in many aspects. Moreover, the data delivery ratio, network throughput, and end-to-end delay are vital performance parameters in designing a multipath routing protocol in WMSNs. In multipath routing, these parameters help to identify a suitable path to forward data to the sink node, since all the discovered paths between the source and sink node have a different quality of service (QoS) value. For time critical and delay sensitive data packets, the protocol needs to direct the traffic in relatively hassle-free routes according to the QoS demands of the application for which the network is deployed.

### 3.2. Energy Model

**Figure 2:**
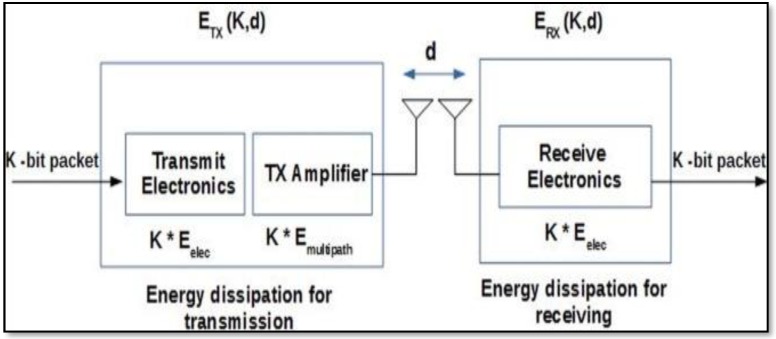
Energy model.

We assume that the multimedia sensor nodes are battery-powered, and the energy of the sensor nodes cannot be replenished. To model the energy consumption of the sensor nodes, we use the same model as in [42],which is diagrammatically shown in Figure 2. Let $E{\left(K,d\right)}_{\mathrm{TX}}$ be the energy consumed by a sensor node, $v_{i}\in V$, to transmit K-bit data to another sensor node, $v_{j}\in V$, that is d distance away in the WMSN. Every time a sensor node, $v_{i}\in V$, transmits sensed data to another sensor node, $v_{j}\in V$, in the WMSN, the sender node consumes a certain amount of energy. This energy depends on whether the transmission is over a single-hop (i.e., direct transmission) or is multi-hop. In the single-hop case, a free-space model ($d^{2}$ power loss) is used, whereas in the multi-hop case a multi-path fading model ($d^{4}$ power loss) is used. Therefore, $E{\left(K,d\right)}_{\mathrm{TX}}$ can be defined as shown in Equation (1):

(1) $$E{\left(K,d\right)}_{\mathrm{TX}}=\;\{\begin{array}{c} K\cdot E_{\mathrm{elec}}+\;K\cdot \varepsilon_{\mathrm{fs}}\cdot d^{2},\;d<d_{0}\\ K\cdot E_{\mathrm{elec}}+\;K\cdot \varepsilon_{\mathrm{mp}}\cdot d^{4},d\geq d_{0} \end{array}$$

where, $E_{\mathrm{elec}}$ is electronics energy, $\varepsilon_{\mathrm{mp}}$ is the multipath energy loss and $\varepsilon_{\mathrm{fs}}$ representsfree space energy loss; $d_{o}$ is a threshold distance and is computed as $d_{0}=\sqrt{\frac{\varepsilon_{\mathrm{fs}}}{\varepsilon_{\mathrm{mp}}}}$. The threshold is used to determine whether the transmission is a multi-hop (i.e., $d\geq d_{0}$) or a single-hop (i.e., direct transmission ($d<d_{0}$)) and assign the appropriate energy consumption based on the transmission distance:

(2) $$E_{\mathrm{RX}}\left(K\right)=K\cdot E_{\mathrm{elec}}$$

Similarly, the energy consumed by a sensor node, $v_{i}\in V$, to receive K-bit data from another sensor node, $v_{j}\in V$, that is d distance away, is computed as the product of the size of data and the energy dissipated by the receiver electronics ($E_{\mathrm{elec}}$) as shown in Equation (2).

### 3.3. Problem Overview

Sensor nodes in WMSNs are battery powered and deployed in an area where charging or replacing the battery is impossible. This situation poses a critical problem in the life of the network [12] and requires a mechanism to minimize energy usage. Therefore, given a set of n battery-poweredmultimedia sensor nodes and a base station (BS), as shown in Figure 1, the problem is to maximize the multimedia wireless sensor network lifetimewhile at the same time minimizing energy usage between the cluster head and base station. This problem can be formulated as follows:

(3) $${\max\;T}_{\mathrm{ntk}},\;\min\underset{i,j\in V\;,\;\left(i,j\right)\in e_{ij}}{\sum }e_{\mathrm{ij}}$$

where, ${\;T}_{\mathrm{ntk}}\;$is the network lifetime and $e_{\mathrm{ij}}$ is the energy consumed by the sensor node in a cluster.

**Theorem** **1.** *The life span of a WMSN mainly depends on the energy of each node in the network. Furthermore, considering the multipath routing protocol, the network lifetime will be directly related to the residual energy and the number of hops along the multiple paths between the cluster head and the sink node.*

**Proof.**  Residual energy of a node, energy consumption, and several hops along a particular path, have a direct association with the lifetime of a network. That is to say, when energy consumption and residual energy of the nodes decreases, the network lifetime proportionally increases.

Let us assume that we have *h* hops in a path, and each hop is supposed to have $E_{\mathrm{CPU}},E_{\mathrm{rec}},{\;\mathrm{and}\;E}_{\mathrm{TX}}$ as energy expenditure by the CPU during computational processing, a packet reception from the predecessor hop, and a packet transmission to the next hop, respectively. The energy consumed in forwarding a packet along a particular path from a CH to the sink can be determined based on the radio energy modeling Equations (1) and (2), which then gives the following Equation (4):

(4) $$E_{path}=\;\overset{h}{\underset{i=1}{\sum }}\left(E_{CPU}+E_{rec}+E_{tx}\right)$$

Suppose all the hops in a particular path have similar energy expenditure and we prove our hypothesis about the association between the lifetime of a network and the remaining energy of a hop, the energy dissipated, and several hops along the path. Similarly, we have:

(5) $$E_{path}=h*\left(E_{CPU}+E_{rec}+E_{tx}\right)$$

However, the energy consumed by the processor is much less than the energy consumed by the transceiver for receiving and transmitting a K-bit data packet as shown in Equation (6):

(6) $$E_{CPU}\ll E_{rec}+E_{tx}$$

Therefore, we have Equation (7) from Equations (5) and (6):

(7) $$E_{\mathrm{path}}=h*\left(E_{\mathrm{rec}}+E_{\mathrm{tx}}\right)$$

We can deduce the following from Equation (7):

(8) $$E_{rec}+E_{tx}=\;\frac{E_{path}}{h}$$

A particular path between a CH and the sink serves only until the energy level of a particular hop along the path has the least required energy. Otherwise, the failure of one or more nodes along the path will result in breaking up the path. The minimum energy of a node in the path can be used to partially determine the minimum working time of the path. Similarly, the minimum working time for a hop in a path *p*, $T_{p}$ can be determined as shown in Equation (9):

(9) $$T_{p}=\;\frac{E_{min}\left(p\right)}{E_{rec}+E_{tx}}$$

From Equations (8) and (9):

(10) $$T_{ntk}=\;\frac{E_{min}\left(p\right)}{E_{path}}*h$$

In most literature, the lifetime of a particular WMSN is defined based on “first node die” (FND). Hence, the minimum network lifetime will also be associated with the minimum hop energy, as shown in Equation (11).

(11) $$T_{ntk}=\underset{i\in h}{min}\;T_{p}$$

(12) $$T_{ntk}=\underset{i\in h}{min}\frac{E_{min}\left(p\right)}{E_{path}}*h$$

Thus, $T_{ntk}$ is directly associated with the energy consumption in a path, the residual energy of the minimum energy node, and the hop distance to the base station.

When combining Equations (3) and (12), maximizing the minimum time utilized in a path will maximize the overall network lifetime as shown below in Equation (13).

(13) $$\mathrm{maximize}\;\underset{p\epsilon h}{\min}\;T_{p}$$

From Equation (13), we can see that the network lifetime of a WMSN has a direct relationship with the minimum residual energy of a hop in the path and the hop count (path distance). Moreover, it has an inverse relationship with the total energy consumption of the path.  □

## 4. Energy Efficient GA-Based Multipath Routing

**Figure 3:**
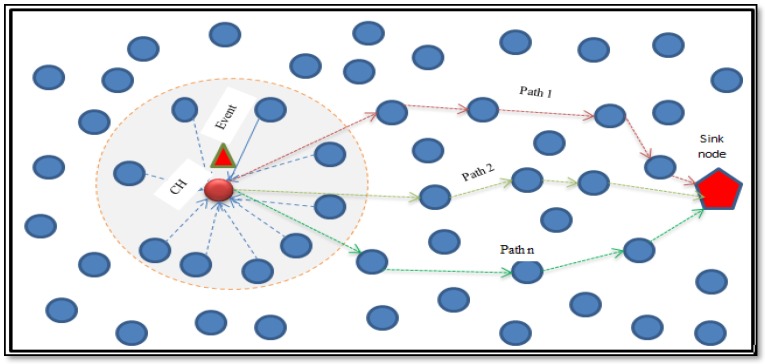
Efficient multipath routing based on genetic algorithm (EMRGA).

In this section, we present the proposed multipath routing for a wireless multimedia sensor network. We refer to the proposed algorithm as efficient multipath routing based on GA (EMRGA). As shown in Figure 3, the proposed routing protocol is a cluster-based multipath framework that allows message transfer between the cluster heads and the base station. The main aim is to minimize the energy consumption as well as the overhead due to routing. We describe the dynamic cluster formation, the cluster head selection, and the multipath formation for data communication in detail below.

### 4.1. Cluster and Cluster Head Formation

To ensure scalability, save the battery life of the individual multimedia wireless sensors, and to prolong the network lifetime, the sensor nodes in the wireless multimedia sensor network are divided into a set of clusters [43], where each cluster is managed by a cluster head (CH). Algorithm 1 shows the procedure used to create a cluster. The cluster is formed dynamically. All nearby nodes whose received signal strength are beyond a given threshold value or nodes will form a cluster dynamically. The clusters are dynamically formed by the sensor nodes available in an area where a particular event occurs. Those nodes which are in the vicinity of event radius are included in the clustering. One node with better cost value will be selected among the nodes in the cluster as the CH.

**Algorithm 1:** Cluster Formation1: Assign delay time $T_{d}$ for every node2: Event radius is *R*3: Calculate $\mathrm{Dist}\left(v_{i},\;\mathrm{Event}\right)$ based on ${\mathrm{RSS}}_{i}$
4: WHILE ($T_{d}\neq$ 0) DO5: IF $({\mathrm{RSS}}_{i}\geq \mathrm{Threshold}$) THEN6:  IF(($\mathrm{Dist}\left(v_{i},\;\mathrm{Event}\right)\leq R$) && (${\mathrm{RSS}}_{i}\geq \mathrm{Threshold}))$
7:   $m_{i}=m_{i}+1;$ // number of nodes in the cluster8:  END9: END10: END 

Once the cluster members are determined, the next step is to select a cluster head. The detail of the cluster formation algorithm is described as Algorithm 2.The algorithm used to select the CH can be explained as follows. Suppose we have m sensor nodes and let $X=\left\{x_{1},x_{2},\;\;x_{3},\;x_{4},\;\ldots ,\;x_{m}\right\}$ be the sensor nodes positions in the event area respectively. In the first round, the node positioned relatively in the middle of the cluster will be the cluster head. In subsequent rounds, the center of the event-region will be determined by computing its centroid and comparing the value with the position of each node in the cluster. A node which is relatively closer to the centroid of the event-region will be the CH.

**Algorithm 2:** Selection of Cluster Head
**BEGIN**
1: INPUT: *m* nodes in a cluster2: **FOR *i***←**1: *m***3: $v_{i}$ calculates its cost as $\cos t\;\left(v_{i}\right)$4: **END-FOR**5: Assign delay time $T_{d}$ for every non-CH node in the cluster6: **WHILE (**$T_{d}\neq 0)$
7: wait $T_{d}$ moreover, all nodes in the cluster receive CH request from other members;8: Assign the node with higher cost value to be CH9: **END-WHILE**10: CH sends an advertisement message to other nodes in the cluster;11: Connect CH with other remaining m−1 non-CH members for data communication12: **END**

The cluster members send their sensed data to the cluster head during their allocated time. The cluster head, in turn, collects these data and aggregates them before forwarding to the sink node. The aggregated data then are continuously transmitted to the base station until the event stops. As the cluster head performs extra work, it uses more energy compared to the other nodes in the cluster. To avoid premature death of any sensor nodes in the cluster, the cluster head responsibility is shared among the cluster members by switching the role between them. The algorithm considers the transmission energy cost, residual energy, distance between source nodes and CH, and distance between CH and base station. Specifically, a new CH is selected by computing the cost of the nodes as shown in Equation (14). Therefore, in each subsequent round, a node which has a higher cost value will be elected as CH.

(14) $$\mathrm{Cos}t\left(v_{i}\right)=\;\frac{\mathrm{Dist}\left(v_{i},{\;C}_{e}\right)}{m}+\;\frac{\mathrm{Dist}\left(v_{i},\;\mathrm{BS}\right)}{m}+E_{\mathrm{res}}\left(v_{i}\right)$$

where $Dist\left(v_{i},\;C_{e}\right)$ is the distance between a node, $v_{i}$, and, the center of the event area, and $\mathrm{Dist}\left(v_{i},\;\mathrm{BS}\right)$ is thedistance between a node, $v_{i}$, and the base station (BS).${\;E}_{\mathrm{res}}\left(v_{i}\right)$ is the residual energy of the node. This function considers the remaining residual energy of the non-CH nodes in the cluster, the distance of each node to the centroid of the event region, and the distance of each node from the sink node. The Euclidian distance between two nodes, $v_{i}{\;\mathrm{and}\;v}_{j}$, in the network can be calculated with the following formula;

(15) $$D_{\mathrm{ij}}=\;\overset{m}{\underset{k=1,k\neq j}{\sum }}{\left(x_{i}-x_{j}\right)}^{2\;}+\;{\left(y_{i}-y_{j}\right)}^{2}$$

In general, a node placed near the center of the event has more probability of being the CH. This means that if the node’s distances to the centroid of the event area and to the base station are minimum, then the energy consumed for data transmission will also be reduced.

### 4.2. Multipath Formation Using Genetic Algorithm

The multipath routing algorithm uses a genetic algorithm (GA) to find multiple paths in the wireless multimedia sensor network to transfer the data. GA is employed for multipath searching in the network. The best path will be dynamically selected based on the cost function, with minimum distance, and least energy dissipation. The pseudo-code in Algorithm 3 shows the overall steps of the proposed GA-based algorithm.

#### 4.2.1. Encoding of Chromosome

**Figure 4:**
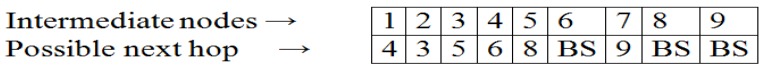
Chromosome representation for multipath.

A routing path from CH to sink node is encoded as a chromosome. This chromosome is encodedas asequence of positive integers, which represent each sensor node found in the path from CH to the base station with length *m*, where *m* is equal to cluster size. By using the gene values, for instance, {4, 3, 5, 6, 8, BS, 9, BS, BS}, we can find the entire paths from every intermediate node to the sink node. For example, let us look in Figure 4 below to understand how the multipath is encoded in the chromosome. If CH’s position is 1, then the path to the base station will be CH→4→6→BS, or if the CH is placed at position three, then the path will be CH→5→8→BS.

A group of the chromosome is called the population. To begin with, we have initialized the population with the size of the cluster. The validity of a chromosome is assured if two things are satisfied; firstly, if all possible multi-paths in the chromosome donot have any loop, and secondly, if the gene value in the chromosome is among one of the valid possible next hops in the network.

#### 4.2.2. Fitness Function

The fitness function calculates the most energy efficient path. The appropriateness and quality of a particular solution for a given problem should be measured by checking its fitness among the other possible options by employing a fitness function. In this paper, we have used the fitness function illustrated below in Equation (16). The fitness function is based on maximizing the cost value as described in Equation (17) to find an energy-efficient path to the base station. This function considers the minimum distance to both the sink node and the immediate next connecting node, and the remaining energy of the next hop:

(16) $$\max f\left(v_{i}\right)$$

where,

(17) $$f\;\left(v_{i}\right)=\;\frac{1}{\mathrm{Dist}\left(v_{\mathrm{source}},{\;v}_{i}\right)}+\;\frac{1}{\mathrm{Dist}\left(v_{i},\;\mathrm{BS}\right)}+E_{\mathrm{res}}\left(v_{i}\right)$$

where $\left(v_{i}\right)$ is the fitness value of the next possible node from the current node, $E_{\mathrm{res}}\left(v_{i}\right)$ is the remaining energy of the node $v_{i}$, $Dist\left(v_{source},\;v_{i}\right)$ moreover, $Dist\left(v_{i},\;BS\right)$, using Equation (15).

The next hop in the path is selected if it has the maximum fitness value among the possible nodes. Maximum cost value for the fitness function will be obtained if both distances (i.e., $\mathrm{Dist}\left(v_{\mathrm{source}},{\;v}_{i}\right)\;$and $\mathrm{Dist}\left(v_{i},\;\mathrm{BS}\right)$) have minimum values, and the next hop has higher residual energy than other possible candidates. In the next step, a chromosome with a fitness value higher than the others will be selected for the next process called crossover. Here, we use Roulette-Wheel [43] selection as this model works better for our problem.

#### 4.2.3. Crossover

In the crossover process, the parent chromosomes, which are selected based on their fitness value, will swap their gene position. The newly generated child chromosomes will be evaluated based on the fitness function. If their fitness value is greater than that of their parents, then the parent chromosomes will be replaced by their offspring chromosome and will be discarded, or vice versa.

#### 4.2.4. Mutation

In this study, we applied the model explained in Equation (18) for the mutation process. The model acquires three randomly chosen genes from the parent chromosomes. A random number, α∈(0, 1), is chosen for calculating the new position of the mutated gene. At the end of the mutation process, the fitness value of the new mutated genes is evaluated. If it is found fit or valid, then it will be inserted in to the current population. Finally, it will be passed to the next phase. The position of new gene:

(18) $$\left(x,y\right)=\left(x_{1},y_{1}\right)+\alpha *(\left(x_{2},{\;y}_{2}\right)-\left(x_{3},{\;y}_{3}\right)$$

where (x,y) is the new gene position in a chromosome, $\left(x_{n},y_{n}\right)$ *n* = 1, 2, 3 are three randomly chosen parent gene positions, and 0≤α≤1.

**Algorithm 3:** Genetic AlgorithmBEGIN1: Initialize population with equal to cluster size2: Determine the fitness value for each sensor node3: REPEAT4: Select parent chromosomes from the population5: Perform Crossover operation to produce a new child6: Perform mutation7: Compute the fitness of each mutated individual8:    **IF** (Child has higher fitness value)9:    Substitute the parents by the new offspring10:  **END**11: END END 

## 5. Performance Analysis

In this section, we discuss the performance analysis of the proposed EMRGA protocol and compare it with several protocols. The experimental setup and the performance metrics used are also explained. The results are also discussed. Table 2 shows the network and GA related parameters with their values. The proposed technique was compared with the TEEN [44], and three multipath algorithms MP [45], MACS [46], and MRP [29],respectively. 

As in [47,48,49], we used MATLAB 2016(a) version running on an HP desktop system with internal processor Intel^®^ Core ™ i7-3770 CPU @ 3.40 GHz with 12GB RAM and 500GB HDD for the simulation. We used Matlab for the modeling environment, including nodes, timing, protocols etc. Briefly, we first created the WMSN by defining its parameters (location, radius, available battery power, etc.). These parameters were stored in different matrices. For each node, we considered that the node died when the node exhausted its available energy. The initial coordinates of the sensor were randomly generated using a Matlab function. The sensor nodes were then placed in the given space based on the randomly generated coordinates. We then connected nodes if the distance between them was less than or equal to the communication radius by creating a bidirectional link between them, each with its costs. We then ran the dynamic clustering mechanism to create the cluster and select the cluster head, and finally coded the routing scheme based on the genetic algorithm. The system runs in rounds and some parameters of the WMSN were calculated such as the time for sending a packet between sensor nodes and the energy consumption. The simulation ended when there was no path to the base station, meaning the cluster heads near the base station were out of energy.

### 5.1. Experimental Setup

We used the following performance metrics to compare the routing protocols:

- *Average Residual Energy***:** This parameter provided the mean remaining node’s energy per round, where n is the number of nodes in the network:
  (19) $$E_{\mathrm{avg}}=\frac{\sum_{i=1}^{n}E_{\mathrm{res}}\left(i\right)}{n}$$
- *Energy Consumption***:** This parameter provided node’s energy dissipation in the event region per round, and m is the number of nodes in the cluster:
  (20) $$E_{\mathrm{cons}}\left(i\right)=\overset{m}{\underset{i=1}{\sum }}E\left(i\right)-E_{\mathrm{TX}}\;\left(i\right))$$
- *Standard deviation (SD)***:** This parameter showed the variation between the individual node’s energy consumption versus the mean energy consumption of the nodes in the network per round. Small SD values signify better load distribution in the network.
  (21) $$\mathrm{SD}=\sqrt{\frac{\sum_{i=1}^{n}{\left(E_{\mathrm{res}}\left(i\right)-E_{\mathrm{avg}}\right)}^{2}}{n}}$$
- *Lifetime of Network***:** This parameter provided the duration for which the network would continue to serve the purpose it wasmadefor, without failing. There are various definitions given for network lifetime by researchers [50,51]. Some researchers define network lifetime as the duration until a certain percentage of nodes die or remain alive: first node die (FND), half node die (HND), and last node die (LND). Similarly, we defined network lifetime as the time the network took for the first node to die (FND).

### 5.2. Discussion of Results

We evaluated the efficiency of the four algorithms in terms of mean residual energy of nodes in the network, energy of consumption of nodes in the event area, the standard deviation of the residual energy of nodes against the mean energy dissipation of the network, and the network lifetime, using FND. We examined the performance of the protocols both under the homogeneous network and under the heterogeneous network. By homogeneous network, we mean the entire sensor nodes had the same initial energy and other characteristics, whereas the heterogeneous network means the nodes had different initial energy.

#### 5.2.1. Homogenous Network

**Figure 5:**
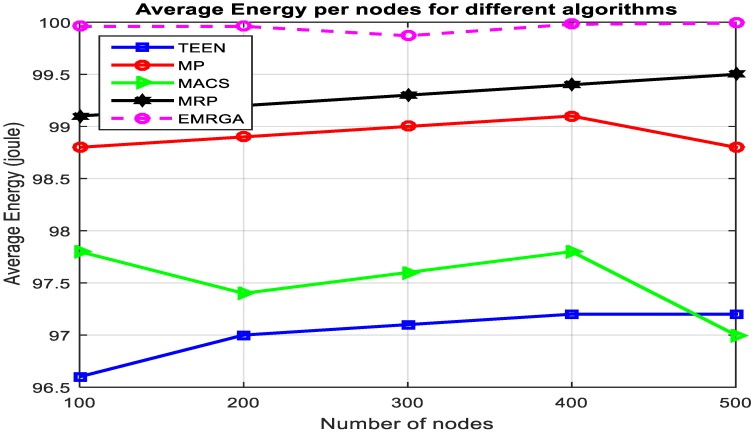
Average residual energy.

**Figure 6:**
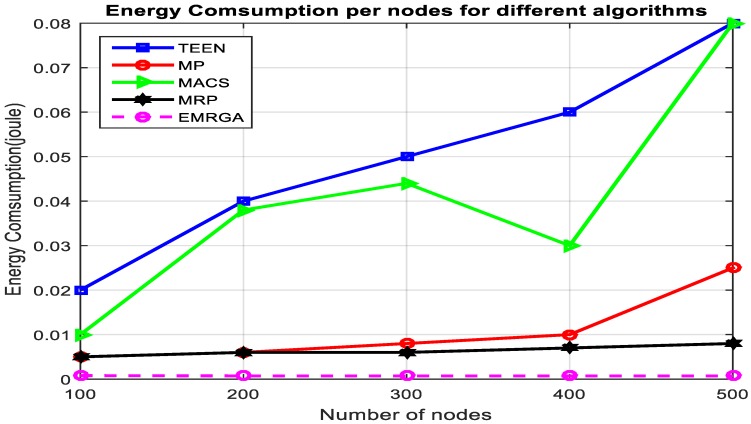
Energy consumption.

**Figure 7:**
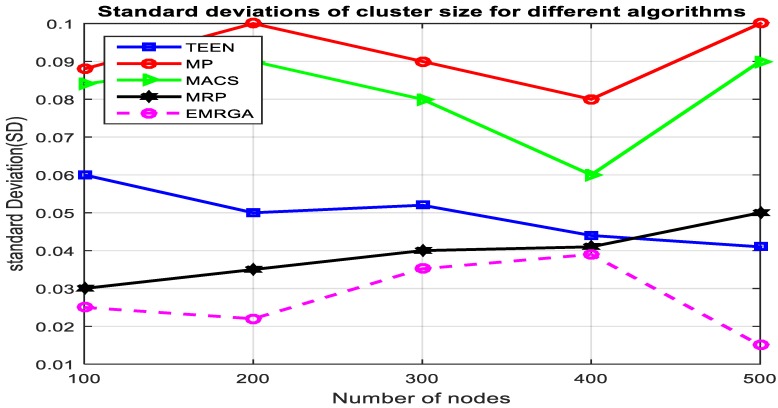
Standard deviations.

**Figure 8:**
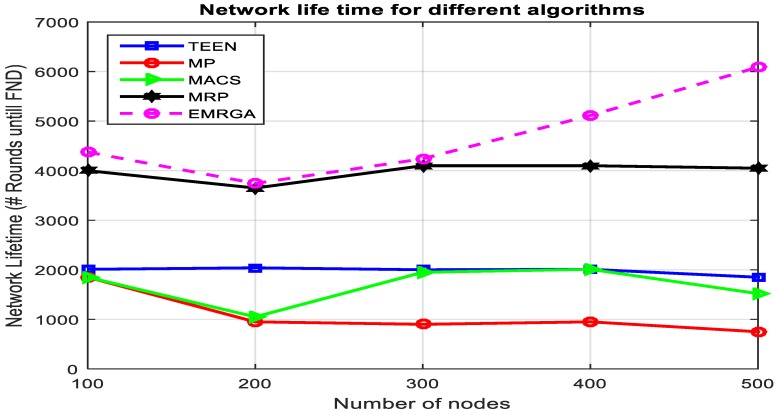
Network lifetime.

In scenario 1, we evaluated and compared the algorithms in a homogeneous sensor network of 100 to 500 nodes, as depicted in Figure 5. The simulation results are shown for the four metrics. EMRGA outperformed the other existing algorithms in all the parameters as illustrated from Figure 5, Figure 6, Figure 7 and Figure 8.

Figure 5 illustrates the mean energy of sensor nodes by applying the four algorithms. EMRGA is observed as having higher average energy than the other protocols. The simulation result revealed that the proposed algorithm consumed less energy for data communication. Though the performance was much higher than the other algorithms in terms of average residual energy, EMRGA showed minor fluctuations when the number of nodes was 300.

In Figure 6, it is observed that in the denser network, the energy consumption of EMRGA also linearly increased. Higher traffic was also observed due to the higher number of nodes in the network. Though MRP and MP both had better energy consumption, the proposed EMRGA still outperformed all the other algorithms for total energy consumption in the network. TEEN uses a dynamic clustering algorithm, but it consumed more energy than EMRGA. In the case of MACS, it had a better energy consumption rate than TEEN. Even though MP is not a hierarchical routing technique (flat), its primary path utilized a small amount of energy in contrast to MACS and TEEN.

According to Figure 7, the standard deviation of energy consumption of each node in the network from the mean energy utilization of the network is lower in the case of the proposed protocol, EMRGA, when compared to other algorithms for all numbers of nodes in the scenario. This provides assurance that it is efficient enough in energy consumption and load balancing across the sensor nodes regardless of the number of nodes in the network. When the number of nodes in the network linearly increased, the SD fluctuated up and down. However, when the network size was greater than 400, the SD decreased continuously ensuring that the energy consumption in the network was maintained at a low level. Moreover, the variation between node energy consumption and overall mean energy dissipation of the network was kept to a minimum which provides assurance that the proposed algorithm maintained load balance across the nodes in the network thereby prolonging the life span of the network.

As depicted in Figure 8, EMRGA showed better, but only slightly improved, maximum network lifetime compared to MRP when there were 300 nodes. However, for more than 300 nodes EMRGA showed significant improvement in the network lifetime compared with the other four algorithms. This ensures that our proposed algorithm was never influenced by the density of nodes in the network. Minimum energy dissipation and better load balancing across the network were achieved when using EMRGA compared with the existing algorithms. This was due to two reasons. The first is because the dynamic clustering technique is employed only when a specific event happens in the network, and the second is due to dynamically selecting the best path using the load balancing function for data delivery.

Even though the energy consumption of MACS was higher than that of TEEN, TEEN’s contribution to prolonging the network lifetime was less than that of MACS. This happened because TEEN employs a clustering technique for data transmission. Out of the five algorithms, MP’s performance on enhancing the sensor network lifetime was the lowest. This is because MP doesnot use alternative paths for data communication and always sends using the primary route to the BS, which rapidly depletes the nodes’ battery energy along the route.

#### 5.2.2. Heterogeneous Network

**Figure 9:**
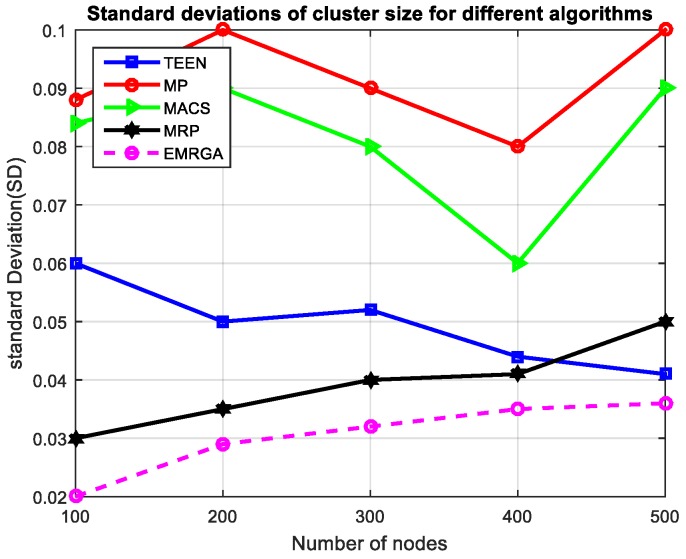
Standarddeviation.

**Figure 10:**
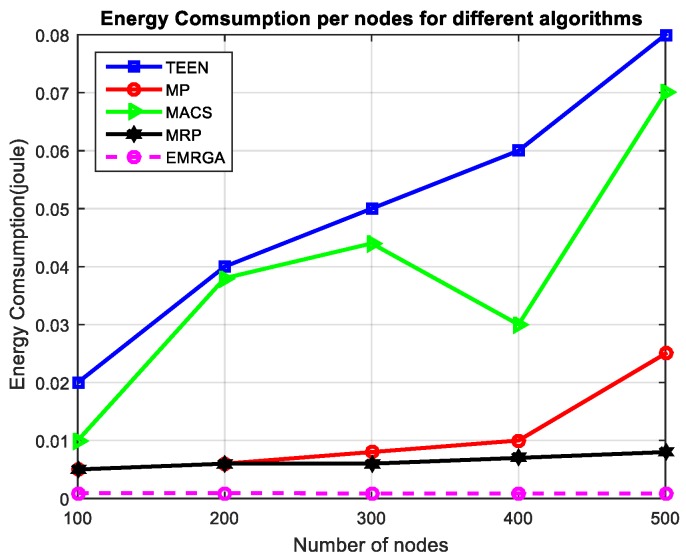
Energy consumption.

**Figure 11:**
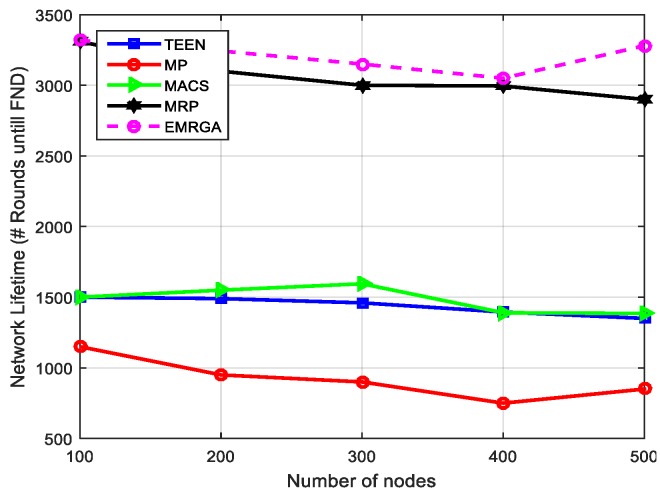
Network lifetime.

**Figure 12:**
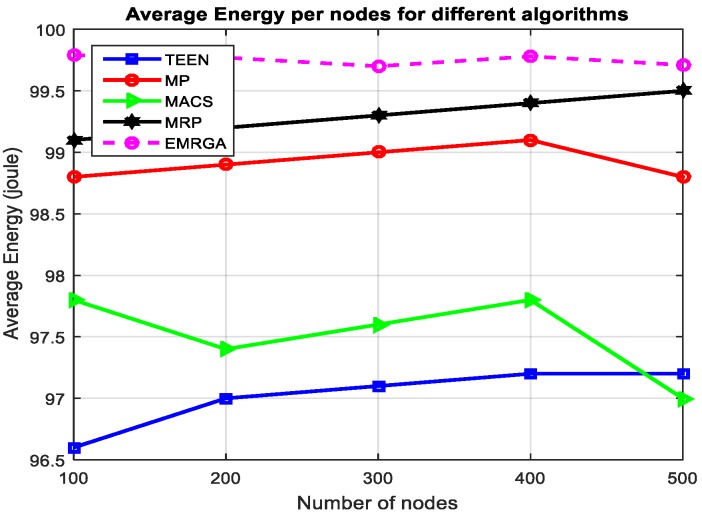
Average energy.

In this scenario, we also compared the performance of TEEN, MACS, MP, MRP, and our proposed technique, EMRGA, using the metrics explained in Section 5.1. In this case, we used a heterogeneous network in which normal nodes were powered with 1 J and advanced nodes with 2 J. We performed five simulations by varying the number of nodes from 100 to 500. The nodes were randomly deployed in the sensor field which was of similar size as in scenario 1. The simulation results are shown below for the four metrics from Figure 9, Figure 10, Figure 11 and Figure 12.

## 6. Conclusions

In this paper, an energy-aware multipath routing technique based on GA wasexplained. We introduced a cost function which considers distance to the center of the event area and base station along with the residual energy of the node for choosing CH, and this improved the energy dissipation of the network. Furthermore, we applied GA for searching for multiple routes for data transmission between CH and the base station. A load balancing function was also used to evaluate and dynamically select the best path. In general, EMRGA maximized the working time of the network by evenly sharing the load across every sensor node. It also minimized the energy consumption of the network compared to existing protocols. This work can be further improved to a network of the same application of mobile sensor nodes with suitable speed.

## Figures and Tables

**Table 1:** The symbols and explanation.

Symbol	Explanation
$v_{i}$	A sensor node in WMSN
$e_{ij}$	The bidirectional wireless link between $v_{i\;}\mathrm{and}\;v_{j}\;$in WMSN
$w_{ij}$	Energy consumed during data transmission between $v_{i}\;\mathrm{and}\;v_{j}\;$in WMSN
$d\left(v_{i},\;v_{j}\right)$	The distance between the node $v_{i}\;\mathrm{and}\;v_{j}$
$S_{event}$	Set of sensor nodes in the event area
***N***	The total number of sensor nodes in the sensing field
***M***	The total number of sensor nodes in the event area
$N_{r}$	The set of neighboring nodes to node $v_{r}$
$d_{o}$	The thresholdvaluetoselect apply transmission model
$\varepsilon_{fs}$	Transmission ability in free space model
$\varepsilon_{mp}$	Transmission ability in multipath fading model
k	Size of data to be transmitted or received by a node
$E{\left(k,d\right)}_{TX}$	Energy consumed to send *k* bits data to a node at a distance d
$E{\left(k\right)}_{RX}$	Energy consumed to receive *k* bits from another node
d	The distance which the data traveled
$E_{cpu}$	Energy consumed for processing data
$E_{elec}$	Electronic energy
$Cost\left(v_{i}\right)$	The overall cost incurred by a node $v_{i}$
$C_{e}$	Center of the event area
$Dist\left(v_{i},\;C_{e}\right)$	Distance between a node $v_{i}$ and the center of the event area
$Dist\left(v_{i},\;BS\right)$	Distance between a node $v_{i}$ and the base station
$E_{res}\left(v_{i}\right)$	Residual energy of a particular node $v_{i}$
$E_{path}$	Energy consumption along a particular routing path between the CH and BS
$E_{min}\left(p\right)$	Minimum energy consumption in path *p*
***H***	Number of hops in a particular routing path between CH and BS
$T_{ntk}$	Network lifetime

**Table 2:** Experimental parameters.

Parameters	Value
Sink	1
Sink position	(0,200)
Sensor nodes	100
Sensing area	200 m × 200 m
Event radius	20 m
Transmission range	200 m
Packet size	512 bytes
Message size	10 bytes
Sensor initial energy (normal nodes)	2 J (1 J for heterogeneous case)
Advanced sensor node initial energy	2 J
$E_{DA}$, $E_{elec}$	5 nJ
$E_{fs}$	10 pJ
$E_{mp}$	0.0013 pJ
Population size	100
Crossover probability	20%
Mutation probability	10%
